# Effects of Glycosylation on the Enzymatic Activity and Mechanisms of Proteases

**DOI:** 10.3390/ijms17121969

**Published:** 2016-11-25

**Authors:** Peter Goettig

**Affiliations:** Structural Biology Group, Faculty of Molecular Biology, University of Salzburg, Billrothstrasse 11, 5020 Salzburg, Austria; peter.goettig@sbg.ac.at; Tel.: +43-662-8044-7283; Fax: +43-662-8044-7209

**Keywords:** secreted protease, sequon, *N*-glycosylation, *O*-glycosylation, core glycan, enzyme kinetics, substrate recognition, flexible loops, Michaelis constant, turnover number

## Abstract

Posttranslational modifications are an important feature of most proteases in higher organisms, such as the conversion of inactive zymogens into active proteases. To date, little information is available on the role of glycosylation and functional implications for secreted proteases. Besides a stabilizing effect and protection against proteolysis, several proteases show a significant influence of glycosylation on the catalytic activity. Glycans can alter the substrate recognition, the specificity and binding affinity, as well as the turnover rates. However, there is currently no known general pattern, since glycosylation can have both stimulating and inhibiting effects on activity. Thus, a comparative analysis of individual cases with sufficient enzyme kinetic and structural data is a first approach to describe mechanistic principles that govern the effects of glycosylation on the function of proteases. The understanding of glycan functions becomes highly significant in proteomic and glycomic studies, which demonstrated that cancer-associated proteases, such as kallikrein-related peptidase 3, exhibit strongly altered glycosylation patterns in pathological cases. Such findings can contribute to a variety of future biomedical applications.

## 1. Introduction

Glycosylation is a posttranslational modification that is found on about 50% of all proteins, in particular on secreted and transmembrane proteins of eukaryotes, archaea and to a lesser extent in prokaryotes [1,2,3]. Eukaryotic proteins require glycosylation for proper folding, oligomerization and solubility, while glycans significantly prolong the stability and half-life time in many cases by protection against proteolysis [4,5]. Although *N*-glycosylation is more frequent, *O*-glycosylation can similarly protect against general and specific proteolysis [6,7,8]. Protein trafficking, i.e., the sending of proteins to cellular compartments or to the extracellular matrix, depends on specific, covalently linked glycans [9]. In addition, glycans play an important role in the interaction and recognition of proteins, such as in the context of immunity and cell adhesion [10,11,12]. Glycosylation may even protect against molecular damage by free radicals [13]. In recent years, increasing evidence was found that glycans have distinct effects on the activity of many enzymes, in particular as regulatory modules for substrate binding and turnover. This study gives an overview on the most relevant types of glycosylation of proteases, regarding the structural knowledge and the functions of glycans. The importance of this little investigated field lies in the enormous diversity of possible glycosylation variants and the altered functionality of proteins under healthy or disease conditions.

**Figure 1:**
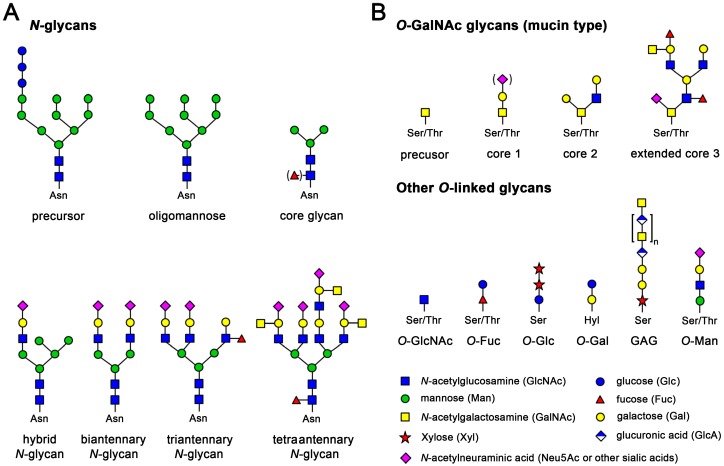
Examples of the most relevant types of glycosylation according to the literature [3,27]. (**A**) *N*-glycosylation of asparagine in sequons with the consensus sequence Asn-Xaa-Ser/Thr. *N*-glycans are generated by trimming and extending the common precursor GlcNAc_2_Man_9_Glu_3_. Small core glycans are mostly intermediates in mammalian glycan synthesis, but often occur in more primitive eukaryotes and insects, as used for recombinant expression. Mammalian *N*-glycans exhibit an enormous diversity, due to many possible combinations of branching sugars; (**B**) *O*-glycosylation at Ser and Thr is found in all kingdoms of life. There is no distinct consensus sequence, but proline-rich regions are favored, e.g., a typical *O*-glycan site would be Pro-Ser/Thr-Xaa-Yaa-Pro. A very common mammalian *O*-glycan is the mucin-type that starts with GalNAc and is extended by galactose and sialic acids or GlcNAc, with eight different cores known. In addition, the *O*-xylose linked, non-branched glucosamine glycans (GAG) or proteoglycans are a large and diverse glycan family. The displayed chondroitin can be phosphorylated and heavily sulfated, comprising up to fifty disaccharide units. *O*-GlcNAc glycans occur inside cells, even in the nucleus, while *O*-galactosylation is found at hydroxylysine residues (Hyl) of collagens.

*N*-glycosylation at sequons of the Asn-Xaa-Ser/Thr type is widespread in proteins of archaea and eukaryotes, whereby proline is largely excluded as Xaa and disfavored as residue following Ser/Thr [14,15]. Some rare sequons are Asn-Xaa-Cys (1%), Asn-Gly (0.5%) and Asn-Xaa-Val (<0.5%) [16]. The process of *N*-glycosylation is extensively described in the literature on glycobiology [17]. Essentially, a newly synthesized polypeptide emerging from a ribosome binds with a signal peptide to a signal recognition particle, which docks to a receptor in the endoplasmic reticulum (ER) membrane and forms a complex with the Sec machinery, which transfers the polypeptide through a transmembrane channel into the lumen of the ER [18,19]. A signal peptidase cleaves the N-terminal signal peptide and the oligosaccharyltransferase complex attaches a GlcNAc_2_Man_9_Glc_3_ precursor at a suitable sequon of the Asn-Xaa-Ser/Thr type [20]. Subsequently, the *N*-glycosylated polypeptide folds in the oxidizing environment of the ER, supported by protein disulfide isomerase for disulfide formation and by various chaperones [21,22]. Afterwards, glucosidases and mannosidases trim the *N*-glycan precursor to Man_5_GlcNAc_2_ or GlcNAcMan_3_GlcNAc_2_ core glycans, which are extended by glucosyltransferases, accompanied by protein quality control and followed by sorting and further processing on their way through the ER Golgi intermediate compartment into the Golgi [23,24]. Final modifications of the *N*-glycans in the Golgi comprise extensions by transferases that attach *N*-acetyl-glucosamine (GlcNAc), fucose, galactose, mannose, and sialic acid sugars, before sorting to secretory vesicles [25]. Variations of branching generate a large diversity of *N*-glycans with distinct composition under physiological and pathological conditions (Figure 1A) [26].

Earlier structural database analyses reported a relatively low percentage of only 27% *N*-glycosylation of all sequons in human proteins, of which 96% belonged to secreted and membrane proteins and 4% to cytoplasmic and nuclear proteins [28,29]. More recent data suggested around 85% occupancy of sequons, with 50% glycosylation of all proteins in the SWISS-PROT sequence data bank [2]. Thorough mass spectrometric analyses demonstrated that in the mouse *N*-glycoproteome the majority of identified sequons is occupied, e.g., 99% of predicted membrane protein *N*-glycosylation sites [16]. Glycosylated Asn residues in human proteins are preferentially located in turns (78%) compared to β-sheets (12%) and α-helices (10%), which resembles the situation in murine proteins [16,28]. The strict structural constraints for *N*-glycosylation are reflected by a similar localization in proteins of fish, insects, plants and lower eukaryotes [28,30].

About 12% of all glycosylated proteins are exclusively *O*-glycosylated, while about 10% of them are both *N*- and *O*-glycosylated [2]. Seven types of *O*-glycosylation have been found in humans (Figure 1B). The mucin-type with *N*-acetylgalactosamine (GalNAc) linked to Ser or Thr in membrane or secreted proteins is more common than *O*-glycosylation with xylose, galactose, glucose, fucose, and mannose, whereas mostly proteins with *O*-linked GlcNAc localize to the cytoplasm and the nucleus [27]. Although no distinct consensus sequence is known, Pro-rich sequences are favored, such as Pro-Ser/Thr-Pro-Xaa-Pro [31,32]. Usually, the first step of *O*-glycosylation of the mucin-type is performed in the Golgi by a GalNAc transferase, followed by extensions and branching, which results in eight core glycan subtypes [27,33]. *O*-glycosylation of the mucin-type is important for tissue development and immune reactions [34].

Among the rarer types of glycosylation is *O*-mannosylation for quality control and protection against proteolysis [35]. Similarly, *O*-glycosylation of hydroxylysine can occur in mammals, in contrast to *O*-glycosylation of hydroxyproline, which is found in other eukaryotes [36,37]. The uncommon *C*-mannosylation of Trp residues plays a role in protein folding, secretion and signaling [38,39]. Glycosylphosphatidyl-inositol-anchored proteins (GPI-APs) are linked to glycolipids as membrane anchors, which is sometimes termed glypation [9,40]. By contrast, glycation is the uncatalyzed covalent linkage of glucose or fructose with amino groups, which is involved in diabetes and ageing related diseases [41,42]. Apart from this unregulated process, most glycans play very well defined roles in physiology, such as in the regulation of enzymatic activity.

## 2. Glycosylated Proteases

Currently, the UniProtKB database lists about 250 glycosylated human proteases out of more than 700, with changing numbers due to putative or ambiguous classification [43]. They exhibit either experimentally identified or predicted *N*-, *O*-, and *C*-glycosylation sites, using the NetNGlyc and NetOGlyc tools, which are currently applicable to mammalian entries and the mucin-type of *O*-glycans [29,44]. The MEROPS database of proteolytic enzymes includes about 1200 known and putative human proteases, including inactive protease homologs [45].

### 2.1. Aspartic Proteases

An overview of the glycosylated human aspartic proteases shows the 11 confirmed or potentially glycosylated members out of altogether 28, which agrees with their prevalent extracellular localization (Table 1). The prototypic aspartic protease pepsin is not glycosylated, despite its secretion into the stomach, where it cleaves proteins between hydrophobic residues, such as P1-Leu and P1’-Leu according to the Schechter–Berger nomenclature [46]. The related, brain expressed β-secretase 1 (BACE1) is a key player in neuronal regulation and a drug target in Alzheimer´s disease [47]. Although the UniProtKB database annotates BACE1 only as *N*-glycosylated according to manual assertion, a recombinant variant carries *N*-glycans according to mass spectrometry and PNGase F treatment [48].

### 2.2. Cysteine Proteases

Another important enzyme class is formed by the cysteine proteases, of which only 12 out of 158 human members exhibit glycosylation sites, among them several cathepsins (Table 1). This phenomenon can be easily explained by the predominant intracellular location with reducing conditions that are required for the activity of many cysteine proteases. Cysteine cathepsins occur in endolysosomes and under pathological conditions even more often outside cells, where they can be regulated by glycosaminoglycans [49]. For example, the important protease families of the apoptosis- and inflammation-related caspases and of the Ca^2+^-signal mediating calpains do not possess any ascertained glycosylation site [50,51]. However, legumain, a monomeric caspase-like cysteine protease, is found in the acidic lysosomes and outside cells, carrying at least three *N*-glycans in its recombinant form, which was generated by *Leishmania* expression [52,53].

### 2.3. Metalloproteases

The mostly Zn^2+^-dependent metalloproteases are secreted from cells to a large extent, with 108 of 163 members carrying at least one glycan (Table 2). Among them are the extracellularly located, soluble or membrane-anchored matrix metalloproteinases (MMPs), which play important roles in development, wound healing, brain processes, and cancer [54]. Their major task is the degradation of matrix proteins by cleaving at larger hydrophobic P1′ residues, which can be supported by collagen-binding hemopexin domains [55]. The majority of MMPs is *N*-glycosylated, although six of them appear to be non-glycosylated, while MMP-25 is potentially GPI-anchored [56]. Several MMPs possess *N*-glycosylation sites, which are required for protein interaction and secretion as observed for MMP-9 [57]. MMP-9 is the remarkable example of a protease with a heavily *O*-glycosylated, Pro-rich linker domain, containing more than a dozen branched mucin-type core 2 glycans [58]. Intriguingly, tumor cells produce MMP-9 mainly with *O*-glycans of mucin core 1, which reduces the binding affinity to its ligand galectin-3 [59]. The two families of transmembrane and GPI-anchored ADAM and ADAMTS proteases, which cleave extracellular portions of other membrane proteins and matrix proteins, are involved in cell adhesion, growth factor shedding, cell migration, morpho-genesis and cancer [60,61]. An outstanding example is ADAM17 or tumor necrosis factor α converting enzyme (TACE), which sheds ectodomains of numerous substrates depending on *O*-glycosylation of cleavage sites [62]. Similarly, the heavily *N*-glycosylated meprin-α and -β act as sheddases and cleave pro-inflammatory cytokines [63,64]. The membrane-bound meprin-β subunits tend to form dimers, whereas α and β subunits can form heterodimers and α subunits may form even higher oligomers [65]. Metallocarboxypeptidases are another relevant subgroup of secreted neurohormone and cytokine processing enzymes, which have been in implicated in diseases of the pancreas, diabetes and cancer and are considered nowadays as promising drug targets [66,67]. To date, the developmentally and pathophysiologically relevant Wnt-signal regulating Tiki 1 and 2 proteases belong to a superfamily of hardly characterized, unique Co^2+^/Mn^2+^-dependent enzymes [68].

### 2.4. Serine Proteases

Serine proteases constitute another large group with about 106 glycosylated members out of 144 according to UniProtKB, with family S1 being the largest one (Table 3). The prototypic digestive proteases trypsin, chymotrypsin and elastase are produced in the pancreas and define the corresponding major substrate specificities, referring to the S1 subsites. Among the digestive proteases, only the membrane-anchored enteropeptidase, also known as enterokinase, is heavily *N*-glycosylated and activates trypsinogen, which in turn activates other digestive enzymes [69]. The blood coagulation cascade involves several trypsin-like proteases, which leads to the activation of prothrombin by factor Xa, resulting eventually in thrombin cleavage of fibrinogen and formation of fibrin clots, which stop bleeding [70]. *N*-glycosylated variants of factor VII propeptides prolong its half-life significantly, while corresponding variants of factor IX can also play a role in the treatment of hemophilia [71,72]. Regulation of the fibrinolytic plasmin depends on differential *N*- and *O*-glycosylation, which alters the structure significantly [73,74]. In addition, both the tissue-type plasminogen activator (tPA) and urokinase-type plasminogen activator (uPA) possess *N*- and *O*-glycans, whose roles have been partially elucidated [75,76]. Kallikrein 1 (KLK1) and the other fourteen kallikrein-related peptidases (KLKs) fulfill numerous crucial tasks in tissue development, reproduction, wound healing or neuronal processes [77]. Since several KLKs exhibit altered expression in various cancers, they are used as biomarkers, e.g., KLK3/PSA (prostate specific antigen), and investigated as drug targets [78,79]. Moreover, most human KLKs are *N*-glycosylated proteins (except KLK14), for which potential functional roles have been described [80,81].

Secretory proprotein convertases (PPCs), among them the *N*-glycosylated furin and PPCs 4, 5, 6, 7, and 9, process many substrates upon secretion (Table 4) [82]. Up to now, no distinct functions of their *N*-glycans were reported, whereas *O*-glycosylation near the cleavage sites of their substrates contributes to the regulation of turnover [83]. As observed for many glycoproteins, *N*-glycosylation is critical for folding, secretion and stability of tripeptidyl peptidase I [84]. Interestingly, the hereditary disease late infantile neuronal ceroid lipofuscinosis is caused by a mutation resulting in the loss of an *N*-glycan and all enzymatic activity [85]. The α/β-hydrolases dipeptidyl peptidase-4 (DPP4) and prolyl endopeptidase (FAP) are multifunctional transmembrane enzymes, which cleave substrates after P1-Pro residues and serve as cell surface receptors [86,87]. Apparently, the heavy *N*-glycosylation of DPP4 does not influence dimerization, activity, and T-cell protection [88].

### 2.5. Threonine Proteases

Threonine proteases are both rare and widespread, due to the central role of the ubiquitin-proteasome system for eukaryotic protein degradation, which is highly conserved from yeast to mammals (Table 4) [89]. Besides 14 inactive α-subunits, the proteasome comprises 14 β-subunits in the two inner rings, of which only six are active and cleave after hydrophobic (β1), acidic (β2), and basic (β5) S1 side chains, which is slightly modified in immunoproteasomes [90]. Proteasomes generate peptides that are presented by most cells as epitopes on MHC class I molecules to cytolytic T-cells [91]. Seemingly, *O*-glycosylation is present in mammalian, but not in yeast 26 proteasomes, while the role of the mucin-type *O*-glycans on four α-subunits and the β1 and probably the β6 subunit remains unclear [92,93].

## 3. Effects of Glycosylation on Proteases

### 3.1. Effects of Glycosylation on Folding, Sub-Cellular Distribution and Secretion of Proteases

Usually, *N*-glycosylation is required for sufficient expression, efficient secretion, and protein trafficking, which holds true for numerous proteases. Glycans facilitate the folding of polypeptides, enhance the solubility of proteins and prevent aggregation [94]. Chymotrypsin C, a pancreatic regulator of trypsin, exhibits two *N*-glycans in the propeptide and the catalytic domain, which serve folding and secretion, but have no impact on activation and activity [95]. A comparison of a fully *N*-glycosylated *Pseudomonas* elastase with its triple mutant Asn43Gln/Asn212Gln/Asn280Gln in the catalytic domain showed virtually no differences in enzyme activity, in contrast to significantly reduced secretion levels [96]. Inspection of this elastase structure (1EZM) revealed that the glycans are remote from the active site, in line with the assumption that the glycans are responsible for proper folding alone as basis for secretion [96,97]. In addition, *N*-glycosylation can be crucial in recombinant expression, as demonstrated for the trypsin-like human KLK5, which could be only expressed in insect cells with a single core *N*-glycan, while expression in *E. coli* cells and subsequent in vitro refolding was not feasible [98]. Similarly, human legumain expression for structure–function studies was largely improved in *Leishmania* cells, which generate short core *N*-glycans [52]. In the case of ADAMTS-9, three *N*-linked glycosylation sites in the propeptide are critical for proper secretion and subcellular localization [99]. Similarly, a mutagenesis study of the human aspartic peptidase renin, which regulates blood pressure by cleavage of angiotensinogen, revealed that *N*-glycosylation at Asn5 and Asn75 plays only a role for secretion [100]. In addition, natural *N*-glycosylation regulates uptake and metabolic clearance, as shown for variants of rat renin [101].

### 3.2. Effects of Glycosylation on Activation and Stability of Proteases

*N*-Glycosylation influences the activation of proteases, both with glycans that are located in the propeptides and in the catalytic domain. Several human proteases possess *N*-glycosylated propeptides, such as KLKs 10 and 13 (Table 3). These glycans may regulate protease activation, similar as in the trypsin-like Der p 3, a dust mite allergen, whose *N*-glycan at position P3 with respect to the cleavage site slows down the activation process [102]. In addition, glycans in the catalytic domain of a proform can be crucial as demonstrated by tunicamycin induced loss of *N*-glycosylation at Asn968 and Asn1087 of rat corin, whose activation failed completely [103]. However, for a recombinant form of the human cysteine protease cathepsin B, no effect of *N*-glycosylation on activation, stability and enzymatic activity was found [104]. Many glycosylation sites protect against proteolysis in sensitive regions, which represents often autoproteolysis of the investigated proteases. For example, the aspartic protease cathepsin E lost rapidly activity and stability when its *N*-glycans were enzymatically cleaved [105]. Enzymatic removal of two *N*-glycans in the 75- and 99-loops from human mast cell chymase did not alter kinetic parameters, only the enzymatic activity decreased faster, suggesting that the glycans protect the exposed surface loops from autoproteolysis [106]. Even the branching of *N*-glycans can contribute to protein stability, as demonstrated for matriptase, which was more resistant to proteolysis by trypsin with fucose β1-6 linked to GlcNAc than with the unbranched *N*-glycan in the protease domain [107].

### 3.3. Effects of Glycosylation on Substrate Binding and Turnover

**Figure 2:**
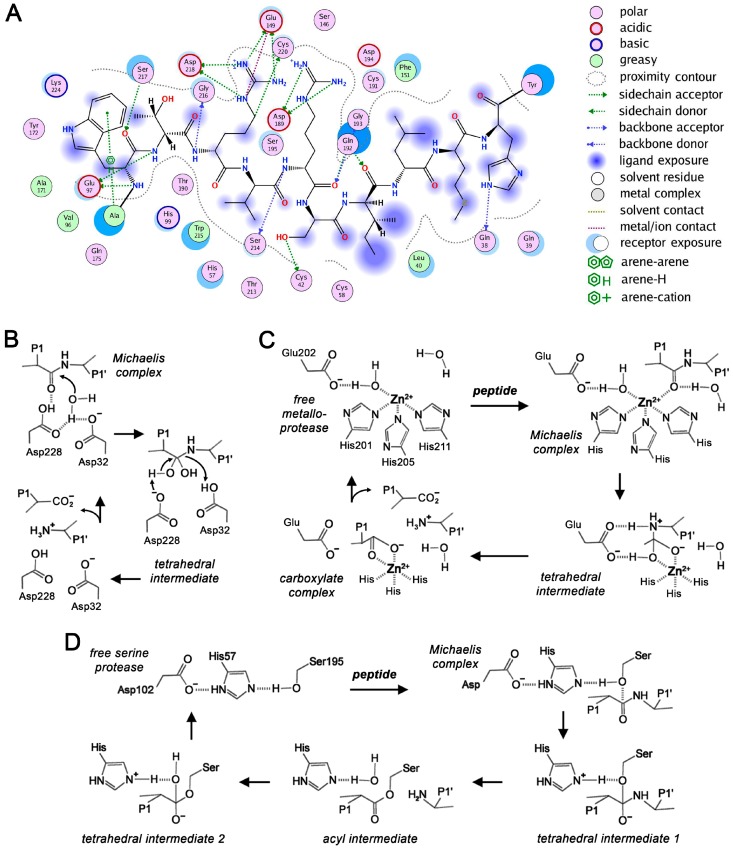
Substrate recognition and catalytic steps in proteases. (**A**) Two-dimensional interaction map of the model peptide AWTRVR-SILMHY with the specificity subsites S6-S6′ of a KLK protease, calculated with the MOE software [114]. The tryptic specificity is based on the electrostatic interaction of P1-Arg and Asp189 in the S1 pocket. Chymotryptic proteases prefer hydrophobic P1 side chains, such as Tyr or Phe; (**B**) The aspartic protease mechanism requires a pair of Asp residues, with one being the general base that activates a water molecule as in BACE-1 [115]. After substrate binding, the nucleophilic water attacks the scissile bond between P1 and P1′ at the carbonyl C atom; (**C**) Three major metalloprotease mechanisms are known, such as the favored one for MMP-3 [116]; and (**D**) Catalysis of a serine protease with chymotrypsinogen numbering [117]. Other serine protease clans exhibit different arrangements of the triad with similar mechanisms. The catalytic triad activates the Ser Oγ as nucleophile via an acid (Asp102) and a general base (His57), which activates a water molecule for hydrolysis of the acyl intermediate [118]. In addition, cysteine protease mechanisms are related, but often require only Cys-His dyads, since the Sγ is more nucleophilic than the Ser Oγ [119]. Similarly, threonine proteases have a nucleophilic Thr Oγ, while the N-terminus and/or a Lys side chain serve as bases [120]. MEROPS lists variations of catalytic residues and rare protease types [45].

Regardless of the protease type, it is more likely that glycans in the vicinity of the active site influence substrate binding in the specificity subsites, which depend on numerous biophysical interactions (Figure 2A). Several examples of aspartic, metallo- and other proteases, which exhibit enzyme substrate (Michaelis) complex and tetrahedral intermediates during catalysis, confirm that the presence or absence of *N*-glycans can considerably alter substrate binding and turnover (Figure 2B–D). Nevertheless, the overall presence or absence of *N*-glycosylation plays no role for the function of some proteases, such as human complement factor I [108].

Numerous examples of *N*- and *O*-glycosylated and non-glycosylated variants of enzymes demonstrate that the presence of glycans can have very diverse effects. Single mutations of the glycosylated Asn632 and Asn651 of the metalloprotease endothelin-converting enzyme had no effect, while the double mutant was completely inactive [109]. A series of sequon mutants of mouse meprin-α showed that six out of ten *N*-glycans are critical for catalysis [110]. The completely deglycosylated snake venom RVV-X, a metalloprotease, which can activate coagulation factor X, exhibited no change in *K*_M_ and a 130-fold reduced *k*_cat_ [111]. Since all *N*-glycans of RVV-X are distant from the active site (PDB code 2E3X) and substrate binding was not hampered, one has to conclude that the overall conformation was significantly affected by their absence [112]. Complete deglycosylation of MT1-MMP (MMP-14) in the *O*-glycan rich linker resulted in a stable and active protease, which, however, failed to bind the tissue inhibitor of metalloproteinases-2 (TIMP-2) and could not form the MT1-MMP/TIMP-2/pro-MMP-2 activation complex [113].

By contrast, removal of glycans enhances the activity of several proteases, most likely because the accessibility of the active site increases. Insect cell expressed ADAM17 with short *N*-glycans has an up to 30-fold increased catalytic efficiency compared to a mammalian cell expressed variant with more complex *N*-glycans, which could interfere with substrate binding [121]. Nevertheless, *O*-glycosylation near the scissile bond of ADAM17 substrates enhances the turnover significantly [122]. *N*-glycosylated plasma kallikrein (KLKB1) has a lower catalytic efficiency than deglycosylated KLKB1, which exhibits an altered cleavage pattern for the substrate insulin [123]. The deletion of a single *N*-glycan in the kringle 2 domain of tPA form II enhanced fibrin binding and the resulting fibrinolytic activity by plasmin [124]. Fully deglycosylated tPA is nearly 4-times more active against small chromogenic substrates, whereby a higher mannose content or removal of sialic acids stimulates the activity as well [125,126]. Since the *O*-glycan in the EGF-like domain and the *N*-glycans in both kringle domains can hardly interfere with small substrates, the glycosylated Asn173 in the 176-loop of the protease domain is most likely critical for the observed effect.

### 3.4. Effects of Glycosylation on Protease Structures

**Figure 3:**
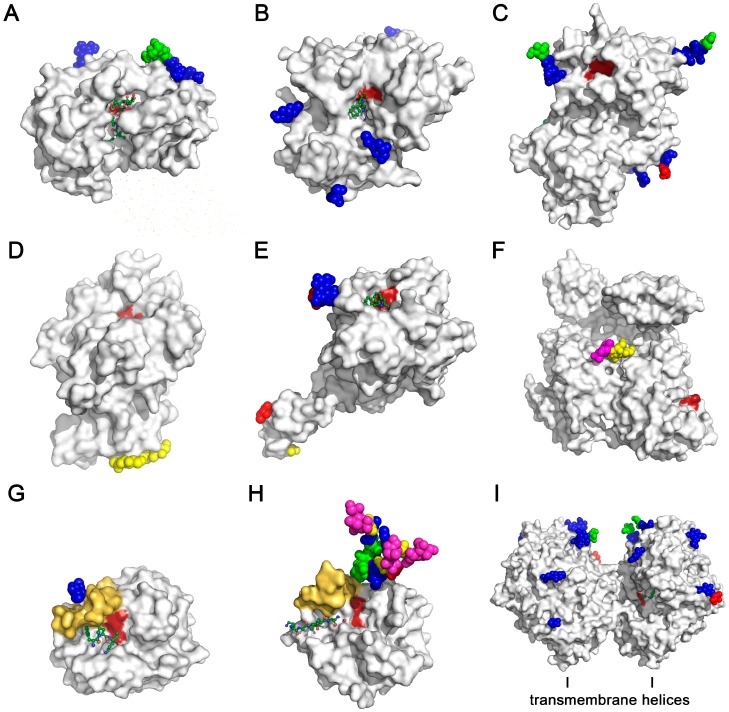
Selected human proteases shown as molecular surface with glycans as spheres according to the standard sugar coloring scheme (Figure 1). Active site residues are colored dark red, while inhibitors or substrates are shown as ball-and-stick models. (**A**) The aspartic protease cathepsin D with two *N*-glycans distant from the active sit cleft (1LYA/1LYB); (**B**) The papain-like cysteine protease cathepsin C has four *N*-glycans, one is located near the substrate binding region (1K3B/2DJF); (**C**) The metalloprotease meprin β carries seven *N*-glycans, with the one at Asn254 located at the substrate binding cleft near the catalytic Zn^2+^ (4GWM); (**D**) Carboxypeptidase N is another Zn^2+^-dependent protease, with three *O*-glycans distant to the active site (2NSM); (**E**) The trypsin-like factor VII is an important activator in the blood coagulation cascade, exhibiting an *N*-glycan in the catalytic domain and two *O*-glycans in the EGF-like domain 1 (1QFK); (**F**) Plasmin, another trypsin-like protease degrades fibrin clots and is *O*-glycosylated between kringle domains 3 and 4 (4DUR); (**G**) The trypsin-like KLK1 exhibits one *N*-linked GlcNAc of a core glycan (1SPJ), which is sufficient to rigidify the flexible 99-loop (yellow) at the active sit cleft, filled with the PPACK inhibitor of the closely related KLK2 (4NFF), in order to explain the effect of glycosylation on substrate binding. In glycan-free KLK2, the 99-loop is more flexible and open; (**H**) The chymotrypsin-like KLK3/PSA carries a triantennary *N*-glycan, which may enhance binding of natural substrates and inhibitors (3QUM/2ZCK). The flexible 99-loop resembles the one of KLK1 (Figure 3G) and is depicted in yellow; (**I**) DPP4 is a membrane anchored, dimeric α/β-hydrolase, with the shielded active site located inside a cavity. The glycans may prevent aggregation and could play a role in the receptor function of DPP4 (1N1M).

Structural studies explain to some extent how glycosylation influences the protein conformation. Small angle X-ray scattering data of an *N*-glycosylated fungal enzyme and its deglycosylated counterpart suggested that the glycans limit the torsion angle range of the polypeptide in general [127]. Currently, only a few studies provide sufficient structural and functional information on glycosylated enzymes and their non-glycosylated variants. Several studies of mammalian lipases, which are closely related to proteases, emphasize the stabilizing role of *N*-glycans, in particular of those close to the active site, for the conformation with the highest activity [128,129,130,131]. Thus, glycans that are located distant from the active site, as seen for the aspartic protease cathepsin D, cannot directly influence substrate recognition and turnover, while these *N*-glycans are important for protein interactions in lysosomal targeting (Figure 3A) [132].

A major consequence of the conformational stabilization conferred by *N*-glycosylation might be an efficient interaction with substrates and regulation of substrate access, which can enhance the specificity. For both cathepsin C and meprin-β a single *N*-glycan at the substrate binding cleft near the catalytic residues appears to be crucial for structure stabilization and substrate binding (Figure 3B,C) [110,133]. Although in some cases the exact role of individual glycans is unclear, it can be deduced that they support oligomerization or interaction with other proteins. The *O*-glycans of recombinant Carboxypeptidase *N* are compatible with substrate binding and tetramerization, however, their function has not been defined yet (Figure 3D) [134]. Double Ala mutants of the *O*-glycosylated Ser52 and Ser60 in the EGF-like domain of coagulation factor VIIa exhibited about 14% of the coagulant activity of wild-type FVIIa, while the amidolytic activity was unchanged, indicating that both *O*-glycans are relevant for the association with tissue factor (Figure 3E) [135]. A comparison of recombinant FVII and plasma FVII confirmed that *N*-glycans with terminal GalNAc instead of sialic acids enhance activity and association with tissue factor [136,137]. Subtle variations of the *O*-glycans at Thr346 (form 1) or Ser248 (form 2) and of the *N*-glycan at Asn288 in plasmin influence binding of the inhibitor α_2_-antiplasmin and the substrate fibrin (Figure 3E) [138,139]. As in several other kallikrein-related peptidases, KLK2 exhibits a single *N*-glycan in the 99-loop close to the substrate binding cleft, which regulates substrate turnover and could increase the specificity for larger protein substrates (Figure 3G) [140]. *N*-Glycosylation in the nearby 62-loop is known for KLK3, thrombin and human neutrophil cathepsin G, where it may modulate substrate binding in the prime side region (Figure 3H) [141]. Eventually, DPP4 is a dimeric transmembrane α/β-hydrolase with various receptor functions beyond its protease activity [142]. As for other integral membrane proteases its *N*-glycosylation may prevent unspecific protein interaction, such as aggregation, but individual glycans may have specific functions (Figure 3I).

### 3.5. Effects of Glycosylation on Protease Mechanisms

**Figure 4:**
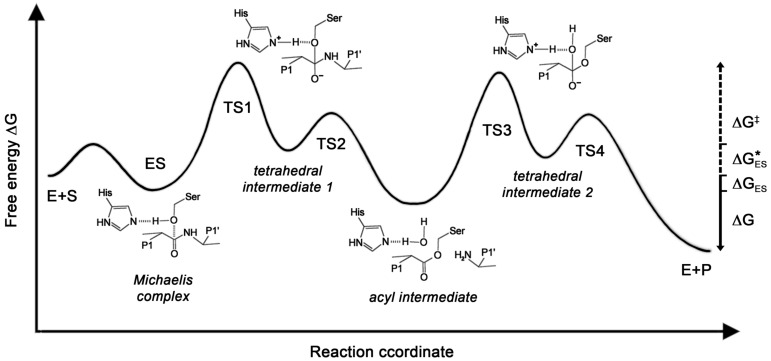
Free energy profile for substrate turnover by the serine protease trypsin [118,144,148]. Various approaches find shifted energy levels and additional energy minima of intermediates and the enzyme product complex [149,150]. The y-axis represents the Gibb´s free energy of the process (∆*G*), which is temperature dependent and related to the reaction enthalpy and entropy: ∆*G* = ∆*H* − *T*∆*S*. The reaction coordinate represents the progress of the reaction, not a real time process. E, S, and P denote enzyme, substrate and products, while TS1 to TS4 are transition states. Direct conversion of *k_cat_* and *K*_M_ into free energy values is not feasible, while they show an inverse correlation, e.g., a high *k*_cat_ with a lower free activation energy ∆*G*^‡^. Currently, the influence of glycosylation on the single mechanistic steps can only be estimated, but differences in the binding energy of the transition states can be calculated as for mutant enzymes [151]. For the substrate Bz-Pro-Phe-Arg-pNA with the *k*_cat_/*K*_M_ values of *N*-glycosylated KLK2 (76,930 M^−1^·s^−1^) and of glycan-free KLK2 (5780 M^−1^·s^−1^) with ∆*G* = −*RT*·ln([*k*_cat_/*K*_M_]_glyc_/[*k*_cat_/*K*_M_]) the result is−6.7 KJ·mol^−1^ [140].

Kinetic and structural data can provide valuable information on enzymatic mechanisms. The basic proteolytic mechanisms are well known and kinetic parameters are available for many substrates, e.g., in the BRENDA database [143]. However, both functional and structural data of glycosylated and non-glycosylated enzymes are scarce. Thus, a model of the possible effects of glycosylation on catalysis by a trypsin-like serine protease shall be described. In general, the Michaelis complex corresponds to the enzyme substrate complex (ES) and the first tetrahedral intermediate is close to the transition state (TS), while the acyl intermediate is the first intermediate product (Figure 2B). The distinct conformational states of these reaction steps were calculated for trypsin with a combined approach of quantum mechanics/molecular mechanics with molecular dynamics/free energy perturbation calculations, resulting in a free energy profile, which was extended by analyzing the role of Asp102 [118,144]. In addition, free energy profiles for the single steps of this mechanism have been determined, including enzyme product complexes, using rate constants from Michaelis−Menten kinetics, viscosity and isotope kinetic parameters (Figure 4) [145]. Although *K*_M_, *k*_cat_, and *k*_cat_/*K*_M_ do not directly correspond to free energies in the reaction profile, they correlate, such as a low *K*_M_ with a high ∆*G*_bind_ of the enzyme substrate complex formation, a high *k*_cat_ with a low ∆*G*^‡^, and *k*_cat_/*K*_M_ with the overall change of the free energy ∆*G* = ∆*G*^‡^ − ∆*G*_ES_ [146,147].

Under certain conditions the free activation energy can be calculated from the catalytic efficiency [152]. Thus, free energy differences between a glycosylated protease and a glycan-free counterpart can be treated as the ones derived from the catalytic efficiency of enzyme mutants [151]. Based on the known catalytic mechanism, such experimental data could be employed to refine the corresponding mechanistic calculations in more detail as shown here.

**Figure 5:**
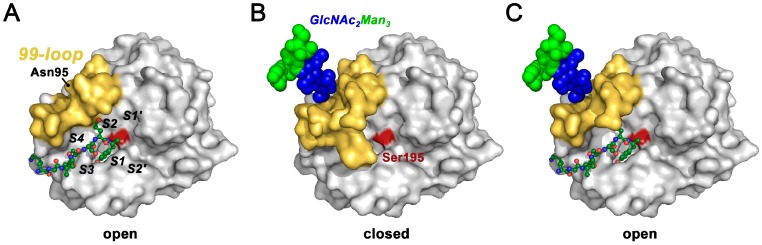
The effect of *N*-glycosylation at Asn95 on the active site conformation of KLK2 according to crystal structure derived models and kinetic data [140]. (**A**) Glycan-free KLK2 expressed in *E. coli* exhibits a wide open 99-loop and access of substrates, depicted as green ball-and-stick model bound to the specificity subsites (S4 to S2′ specificity subsites are labeled); (**B**) *N*-glycosylation at Asn95 favors a closed 99-loop, which covers the non-prime side region, left to Ser195 (dark red) in the standard orientation, which prevents substrate binding; (**C**) The *N*-glycosylated 99-loop of KLK2 opens to a lesser extent than in the glycan-free variant. Thus, substrate binding to glycosylated KLK2 requires more free energy, resulting in a lower *k*_on_ rate and higher *K*_M_.

*N*-glycosylated and glycan-free KLK2 were analyzed with respect to turnover of several small synthetic substrates, resulting always in a 5-fold increased *K*_M_ compared with the *K*_M_ of glycan-free KLK2, while the *k*_cat_ was less affected [140]. This effect could be explained with an influence of the core glycan (GlcNAc_2_Man_3_) at Asn95 in the 99-loop close to the active site, favoring the formation of a type I β-turn over the Asx turn of glycan-free KLK2 [153,154]. Apparently, the flexible 99-loop is wide open in the glycan-free KLK2 crystal structure, whereas it may adopt a closed conformation due to the presence of the *N*-glycan, as in the related KLK1 structure (Figure 3G) [155,156]. The open loop allows for rapid binding of the substrate in the non-prime region at the specificity pockets S4 to S2, which can be explained by a higher *k*_on_ rate, resulting in a lower *K*_M_ = (*k*_off_ + *k*_cat_)/*k*_on_. In addition, substrate binding requires a higher free activation energy ∆*G*_ES_*, since the energy barrier of the *N*-glycosylated, closed loop has to be overcome. Interestingly, the lid-like 99-loop may also serve in fixing the substrate, as observed in a KLK3 acyl intermediate complex structure (Figure 5) [157].

Remarkably, *N*-glycosylated KLK2 was more efficient than the glycan-free variant in autoactivation and to some extent in degrading large protein substrates [140]. This finding can be partially explained by a stronger influence of the 99-loop on small synthetic substrate binding in the non-prime side, but the catalytic efficiency of *N*-glycosylated KLK2 might be enhanced by an optimally shaped active site for stabilizing the transition states. Thus, the decreased ∆*G*^‡^ could depend on an increased *k*_cat_ of the glycosylated enzyme as well. By contrast, *N*-glycans in close to the active site can have a different effect, as shown for porcine pepsin, in which artificial sequons were *N*-glycosylated, resulting in an overall stabilizing effect and a *k*_cat_ decrease to about 30%, which was explained with a more rigid “flap” loop in favorable contact with the S1 and S2 specificity pockets [158]. However, the snake venom thrombin-like enzyme regulates its enzymatic activity, in particular the substrate access to the active site, by a distortion of the *N*-glycosylated 99-loop [159].

Similarly, the presence of the *N*-glycan in the 99-loop of KLK2 appears to regulate the substrate turnover by favoring the closed state (E) over the open E* state, as proposed by the conformational selection model, which is opposed to the induced fit model [155,160]. Thorough analyses of these two mechanistic principles conclude with the combined view of induced fit and conformational selection as extremes of one flux model [161,162]. This model may require further adaptations, as suggested by a molecular dynamics calculation of thrombin carrying a single *N*-glycan at Asn60G, resembling the one of KLK3 (Figure 3H). Seemingly, the *N*-glycan rigidifies distant surface regions that become more flexible upon heparin binding, such as the 62-, 148-, and 176-loops [163]. This phenomenon might be related to the long range interaction between protein sectors, i.e., residues in different segments of hydrolytic enzymes with remarkable functional consequences [164].

## 4. Conclusions and Outlook

Notably, not only the presence of a single glycan can influence kinetic parameters, but its composition as well. In 1988, a groundbreaking NMR study on the glycosylation of porcine, bovine and human plasmin, revealed the positions and composition of the *O*- and *N*-glycans [165]. The now well defined form 1 of human plasmin, in which the *N*-glycan at Asn288 with terminal sialic acids was altered to a high mannose glycan (GlcNAc_2_Man_9_), exhibited a *k*_cat_/*K*_M_ of about 6%, due to interference with substrate binding in the kringle 3 domain (Figure 3F) [139,166]. Glycosylation variants of plasminogen and its activator tPA play a significant role in the activation and activity of the fibrinolytic system [167,168]. Glycans are important for the fine tuning of substrate recognition and binding, as demonstrated by two hybrid plasminogen activator variants, in which the epidermal growth factor-like domain of uPA preceded the kringle 2 and catalytic domain of tPA [169].

**Figure 6:**
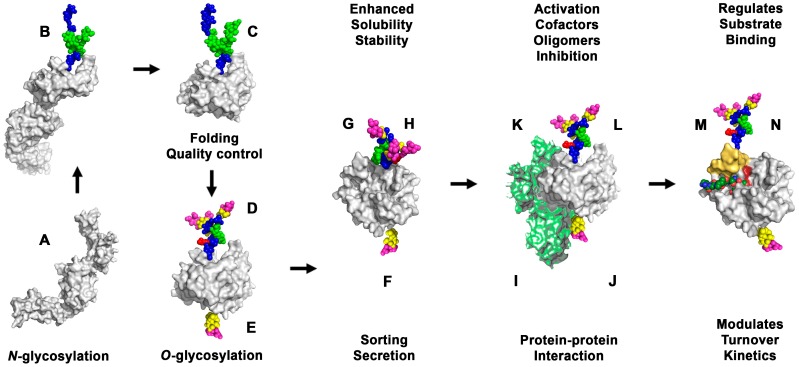
Effects of glycosylation on human proteases. After protein synthesis (**A**) unfolded proteases enter the endoplasmic reticulum (ER), *N*-glycans are linked (**B**); which supports folding (**C**); The *N*-glycans are trimmed and extended, accompanied by quality control (**D**); *N*-glycans are further modified and *O*-glycans are attached in the Golgi (**E**); Sorting leads to membrane anchoring or storage in secretory vesicles (**F**); After secretion, glycosylation prevents aggregation and unspecific binding (**G**); as well as proteolysis, which increases the stability and lifetime of proteases (**H**); Glycosylation regulates binding of: activators (**I**); cofactors (**J**) oligomer partners (**K**); inhibitors (**L**); and substrates (**M**); Eventually, glycosylation fine tunes turnover and kinetic parameters in enzymatic reactions (**N**) [183].

A summarizing overview of the most relevant effects of glycosylation on the physiology of proteases is depicted in Figure 6. Further evidence for the medical relevance of research on glycosylated proteases can be found in the kallikrein field. Natural, inhomogeneously glycosylated KLK3 and glycan-free KLK3 were equally active, recombinant glycosylated KLK3 was three times more active [170,171]. Intriguingly, glycosylation patterns of KLK3 and KLK6 seem to correlate with prostate and ovarian cancer stages [172,173,174]. Mass spectrometry determined forty glycan variants of KLK3 and eleven of KLK6 [174,175,176]. Recently, a glycan-specific immunoassay for cancer-related KLK3 variants has been established [177]. Already a decade ago, cancer-related glycans had been proposed as therapeutic targets [178]. Recently, it was demonstrated that variations of *N*-glycans reflect gene methylation and expression in cancer cells [179]. Altered glycosylation patterns were discovered in other diseases, such as diabetes, and may serve as markers or as targets for future therapies [180]. Biotechnological efforts aim at stabilizing proteases against autodegradation and “humanizing” glycosylation patterns of expression systems for pharmaceutical proteins, in order to abolish unfavorable immunogenic glycan-epitopes [5,181]. The study of effects of glycosylation on protease mechanisms could capitalize on the variation of Cys-linked glycans in the active site, as performed with neoglycoprotein variants of a bacterial subtilisin [182].

Despite the wealth of structural information from X-ray crystallography, this method struggles with glycosylated proteins, since they hamper crystallization and are often too flexible for building models of more than two sugar molecules. Novel methods or old techniques with new applications may facilitate the analysis of glycans on natural proteins, in particular NMR [165,184]. Thus, we are looking forward to a new era of combined efforts from different structural biological methods, which can elucidate the architecture and behavior of naturally glycosylated proteins. Eventually, this knowledge will be highly valuable for the development of better compounds with strongly reduced side-effects and biologically most compatible pharmaceuticals.

## Figures and Tables

**Table 1:** Human aspartic and cysteine proteases with experimentally confirmed or predicted glycosylation sites (*O*-glycosylation indicated in parentheses as “*o*”). MEROPS families are A1, A28 (clan AA), A22 (clan AD), C1, C12 (clan CA), C13 (clan CD), and C26 (PC). UniProtKB entries refer to the first example, following ones continue numerical or alphabetical.Selected structures are shown with Protein Data Bank (PDB) code in bold and numbers of *N*- and *O*-glycans per monomer.

Family	UniProtKB	Aspartic Proteases	PDB (*N-/O-*Glycans)
A1	BACE1_HUMAN	β-Secretase 1, 2	-
	RENI_HUMAN	Renin	**1HRN** (1N)
	CATD_HUMAN	Cathepsin D(*o*), E	D: **1LYA** (2N), **1LYB** (2N)
	NAPSA_HUMAN	Napsin-A	-
A22	SPP2A_HUMAN	Signal peptide peptidase-like 2A, 2B, 2C	-
	HM13_HUMAN	Minor histocompatibility antigen H13	-
A28	APRV1_HUMAN	Retroviral-like aspartic protease 1	-
			
		**Cysteine Proteases**	
C1	CATB_HUMAN	Cathepsin B, C, F, H, K, L2, S, W, Z	C: **1K3B** (4N), **2DJF** (4N)
C12	UCHL1_HUMAN	Ubiquitin C-terminal hydrolase isozyme L1(*o*)	-
C13	LGMN_HUMAN	Legumain	**4AW9** (3N)
C26	GGH_HUMAN	γ-Glutamyl hydrolase	-

**Table 2:** Human metalloproteases with glycosylation sites (*o*: *O*-glycans) according to UniProtKB. MEROPS families are M1, M2, M10, M12, M13, M43 (clan MA), M14 (MC), M16 (ME), M19 (MJ), M20, M28 (MH), M24 (MG) and M50 (MM). M87 and M96 have not been assigned to a clan. UniProtKB entries refer to the first example, following ones continue numerical or alphabetical. Selected structures are shown with PDB code in bold and numbers of *N*- and *O*-sites per monomer. MB: membrane-bound.

Family	UniProtKB	Metallo Proteases	PDB (*N-/O-*Glycans)
M1	AMPE_HUMAN	Aminopeptidase E, N(*o*), Q	E: **4KX7** (8N), N: **4FYT** (8N)
	ERAP1_HUMAN	Endoplasmic reticulum aminopeptidase 1, 2	1: **2YD0** (3N), 2: **5AB0** (8N)
	LCAP_HUMAN	Leucyl-cystinyl aminopeptidase	**4P8Q** (8N)
	TRHDE_HUMAN	TRH-degrading ectoenzyme	-
M2	ACE_HUMAN	Angiotensin-converting enzyme 1, ACE 2	**1O8A** (6N), ACE2: **2AJF** (3N)
M10	MMP1_HUMAN	MMP 1–3, 8, 9(*o*), 12, 13, 14(*o*), 15–17, 19, 21, 23, 26–28	-
M12	ADAM2_HUMAN	ADAM 2, 7–10, 12, 15, 17–23, 28–30, 32, 33	33: **1R55** (1N)
	ADEC1_HUMAN	ADAM DEC1	-
	ATS1_HUMAN	ADAM-TS 1–4, 5(*o*), 6-10, 12, 13(*o*), 14–20	5: **2RJQ** (1N) 13: **3GHN** (3N/1O)
	BMP1_HUMAN	Bone morphogenetic protein 1	-
	TLL1_HUMAN	Tolloid-like protein 1, 2	-
	MEP1A_HUMAN	Meprin A subunit α, β(*o*)	**4GWM** (7N)
M13	ECE1_HUMAN	Endothelin-converting enzyme 1, 2, -like 1	-
	NEP_HUMAN	Neprilysin	**5JMY** (4N)
	KELL_HUMAN	Kell protein	-
	MMEL1_HUMAN	Membrane metallo-endopeptidase-like 1	-
	PHEX_HUMAN	Phosphate-regulating neutral endopeptidase	-
M14	CBPA4_HUMAN	Carboxypeptidase A4, A6, B2, D, E	A4: **2BOA** (1N),B2: **3D68** (4N)
		Carboxypeptidase M, N(*o*), O, X1, Z	M: **1UWY** (1N) , N: **2NSM** (3O)
M16	IDE_HUMAN	Insulin-degrading enzyme	-
M19	DPEP1_HUMAN	Dipeptidase 1, 2, 3	1: **1ITQ** (2N)
M20	P20D1_HUMAN	Carboxypeptidase PM20D1	-
	CNDP1_HUMAN	β-Ala-His dipeptidase	-
M24	MAP2_HUMAN	Methionine aminopeptidase 2(*o*)	-
	XPP2_HUMAN	Xaa-Pro aminopeptidase 2	-
M28	CBPQ_HUMAN	Carboxypeptidase Q	-
	ERMP1_HUMAN	Endoplasmic reticulum metallopeptidase 1	-
	FOLH1_HUMAN	Glutamate carboxypeptidase 2	**2C6C** (6N)
	NALD2_HUMAN	NAALADase 2, NAALADaseL	2: **3FED** (4N), L: **4TWE** (6N)
M43	PAPP1_HUMAN	Pappalysin 1, 2	-
M50	MBTP1_HUMAN	MB transcription factor site protease 1, 2	-
M87	CLCA1_HUMAN	Ca-activated chloride channel regulator 1, 2, 4	-
M96	TIKI1_HUMAN	Metalloprotease TIKI1, TIKI2	-

**Table 3:** Glycosylated human serine protease family S1 (clan PA) members (*o*: *O*-glycans). UniProtKB entries refer to the first example, following ones continue numerical or alphabetical. Selected structures are shown with PDB code in bold and numbers of *N*- or *O*-glycosylation sites per monomer. SP: serine protease.

Family	UniProtKB	Serine Proteases	PDB (*N-/O-*Glycans)
S1	C1R_HUMAN	Complement C1r, C1s, C2 C1r subcomponent-like protein	1r: **1GPZ** (2N), 1s: **1ELV** (1N) 2: **2I6S** (5N)
	CFAB_HUMAN	Complement factor B, I	B: **2OK5** (4N), I: **2XRC** (6N)
	CELA1_HUMAN	Chymotrypsin-like elastase family A1, 3A, 3B	-
	CMA1_HUMAN	Chymase	**1NN6** (2N)
	CORIN_HUMAN	Atrial natriuretic peptide-converting enzyme	-
	CTRC_HUMAN	Chymotrypsin-C, CTR-like protease 1	-
	ELNE_HUMAN	Neutrophil elastase	**1PPG** (2N)
	ENTK_HUMAN	Enteropeptidase (Enterokinase)	
	FA7_HUMAN	Coagulation factor V, VII(*o*), IX(*o*), X(*o*)	7: **1QFK** (1N/2O) 9: **3KCG** (3N)
		Coagulation factor XI, XII(*o*)	11: **5EOK** (4N) 12: **4XE4** (1N)
	GRAA_HUMAN	Granzyme A, B, H, M	B: **1IAU** (2N)
	HABP2_HUMAN	Hyaluronan-binding protein 2	-
	HEPS_HUMAN	Serine protease hepsin	-
	HGFA_HUMAN	Hepatocyte growth factor activator	**2R0L** (1N)
	KLK1_HUMAN	Kallikrein-related peptidase 1(*o*), 2, 3(*o*), 4, 5	1: **1SPJ** (1N) 3: **3QUM** (1N/1O)
		6–13, 15	5: **2PSX** (1N)
	KLKB1_HUMAN	Plasma kallikrein	-
	MASP1_HUMAN	Mannan-binding lectin serine protease 1	**3DEM** (1N)
	NETR_HUMAN	Neurotrypsin	-
	OVCH1_HUMAN	Ovochymase-1, 2	-
	PLMN_HUMAN	Plasminogen(*o*)	**4DUR** (1O)
	POLS2_HUMAN	Polyserase-2	-
	PROC_HUMAN	Vitamin K-dependent protein C(*o*)	-
	PRS23_HUMAN	SP 23, 27, 29, 35, 38, 41, 42, 44, 45, 47, 48, 55–58	57: **4Q7Y** (2N)
	PRSS8_HUMAN	Prostasin	-
	PRTN3_HUMAN	Myeloblastin	**1FUJ** (4N)
	ST14_HUMAN	Matriptase	-
	TEST_HUMAN	Testisin	-
	THRB_HUMAN	Prothrombin	**5E8E** (1N)
	TMPS2_HUMAN	Transmembrane (TM) protease serine 2–7, 9	-
	TMPSC_HUMAN	TM protease serine 12, 13	-
	TM11A_HUMAN	TM protease serine 11A, 11B, 11D, 11E	-
	TPA_HUMAN	Tissue-type plasminogen activator(*o*)	-
	TRYB1_HUMAN	Tryptase α/β-1, β-2, γ, δ	B1: **2F9N** (2N)
	UROK_HUMAN	Urokinase-type plasminogen activator(*o*)	**2FD6** (3N)

**Table 4:** Glycosylated human serine and threonine proteases (*o*: *O*-glycans). Included are families S8, S53 (clan SB), S9, S10, S28 (SC), S60 (SR), T1 and T3 (clan PB) according MEROPS. UniProtKB entries refer to the first example, following ones continue numerical or alphabetical. Selected PDBs with glycans are indicated with numbers of *N*- or *O*-glycosylation sites per monomer. PPC: Proprotein convertase.

Family	UniProtKB	Serine Proteases	PDB (*N-/O-*Glycans)
S8	FURIN_HUMAN	Furin	-
	NEC1_HUMAN	Neuroendocrine convertase 1(*o*), 2	-
	PCSK4_HUMAN	PPC subtilisin/kexin type 4–7, 9	9: **4NE9** (1N)
S9	DPP4_HUMAN	Dipeptidyl peptidase 4	**1N1M** (6)
	SEPR_HUMAN	Prolyl endopeptidase FAP	**1Z68** (5N)
S10	PPGB_HUMAN	Cathepsin A	**4MWS** (3N)
	RISC_HUMAN	Retinoid-inducible serine carboxypeptidase	-
S28	DPP2_HUMAN	Dipeptidyl peptidase	**3JYH** (4N)
	PCP_HUMAN	Lysosomal Pro-X carboxypeptidase	**3N2Z** (5N)
	TSSP_HUMAN	Thymus-specific serine protease	-
S53	TPP1_HUMAN	Tripeptidyl-peptidase 1	**3EE6** (4N)
S60	TRFL_HUMAN	Lactotransferrin	**1LGB** (1N)
			
		**Threonine Proteases**	
T1	PSA1_HUMAN	Proteasome subunit α type-1(*o*), 5–7(*o*)	-
	PSB1_HUMAN	Proteasome subunit β type-1(*o*), 6(*o*)	-
T3	GGT1_HUMAN	γ-Glutamyltransferase 1, 3, 5–7	1: **4GDX** (6N)
